# Neuroethics and treatment without consent

**DOI:** 10.1017/S1092852925000264

**Published:** 2025-04-11

**Authors:** Harry G. Kennedy, Mary Davoren

**Affiliations:** 1 Trinity College Dublin, Dublin, Ireland; 2 Aarhus University, Aarhus, Denmark; 3 University of Bari ‘Aldo Moro’, Bari, Italy; 4 Sapienza University, Rome, Italy

**Keywords:** Neuroethics, psychiatry, treatment, consent, capacity, dignity, best interests, proportionality, compulsion

## Abstract

**Objectives:**

We consider the neuroethics of treatment without consent from a broader perspective than the accepted starting point of functional mental capacities. Notably, in common law jurisdictions, consciousness is seldom admitted in criminal law as a topic for expert evidence of mentalistic defenses or impairments in civil proceedings, yet consciousness and personality are central in Roman law jurisdictions.

**Methods:**

The framework we have adopted is to consider treatment without consent under the headings goals, processes, treatment, and evaluation. The ECHR and the judges of the European Court of Human Rights (ECtHR) are drawn from both common law and Roman law jurisdictions, so that their interpretations and precedents may be informative concerning alternatives to strict application of capacity tests.

**Results:**

There are variable thresholds for treating without consent according to the complexity and amount of information involved, the seriousness of the consequences of untreated illness, the effectiveness of the treatments available and the benefits of earlier intervention, particularly for disease-modifying treatments. Theory-driven principled approaches and scientific medical process approaches to ethical treatment are contrasted.

**Conclusions:**

Carrara’s emphasis on the importance of consciousness and its layered dysfunctions as evidence of competence or impairment appears more robust than a narrow approach based only on functional mental capacity. Capacity—whether general or functional, remains amenable to rules of evidence and legal judgment at the expense of increasingly excessive simplification. Carrara’s emphasis on the inherent dignity of the person appears most in keeping with modern human rights principles.

## Introduction

Treatment without consent has always been an ethical challenge in medicine. In psychiatry and neuroethics, the problem is more complicated, because the person who must make choices and decisions about treatment—the patient, often must do so while impaired in their ability to understand and reason about the decision in hand. We are taking as our starting point, Carrara’s aphorism elsewhere in this volume:

*Together, consciousness and free will form the bedrock of human dignity and liberty, underscoring the responsibility we have towards ourselves and others in crafting a meaningful existence.*

Carrara may be writing from the perspective of ethics grounded in philosophy of mind.^1^ ^,^^2^ We are writing from a medical and common law perspective. In common law, the legal concept of mind has reached a consensus according to which “mind” is defined in law, not as consciousness but more simply as a collection of capacities: to know, including to perceive, to retain and recall, to reason and to make decisions, and then to form intentions and to act, including to communicate the decisions.^3^ ^–^^7^ Current clinical ethics and legal precedents in common law jurisdictions presume free will unless there is evidence to the contrary. We propose that the ability to exercise free will and to accept responsibility for ones acts, omissions, and decisions is a definition of mental health. In order to rebut the presumption of responsibility in criminal law, some impairment of the capacity to form specific intentions must be shown; to rebut the presumption of competence to make decisions about one’s person and treatment, welfare, and financial affairs in civil law, including mental health law, some impairment of mental capacities to make and communicate decisions must be shown. In Roman law, the emphasis is on consciousness, self-awareness, and personality. This is a more holistic approach, but also more hermeneutic, subjective, and unconstrained by readily discernible signs or even symptoms. This contrasts with Wittgenstein’s aphorism “an inner state is in need of an outer sign”.^8^

We also assume that neuroethics is a part of bioethics. The “mind” considered here is embodied and inseparable from anatomical and physiological life and effects in the real world.

In this article we will set out (i) goals as derived from legal and human rights principles. We then consider (ii) decision-making processes. This is followed by examples of practical applications to (iii) treatment decisions. We conclude with a discussion of how to apply (iv) clinical scientific evaluations to consider whether the goals are being achieved by these processes and the duty to achieve excellence through research-driven continuous improvement of health gains for patients.

## Goals

Rather than discuss principles based on human rights, constitutional and legal rights, for this article on neuroethics, we commence by deriving concrete goals (specific, measurable, timed) from such principles. Our objectives are to consider the neuroethics of treatment without consent from a broader perspective and wider context than the routinely accepted starting point of functional mental capacities. Notably, in common law jurisdictions, consciousness is seldom admitted in criminal law as a topic for expert evidence of mentalistic defenses or impairments in civil proceedings. Criminal defenses specifically referring to consciousness, such as automatism and its fashionable variants somnambulism and sexsomnia, the “absence of mind,” are notoriously unreliable.^9^ ^–^^12^ Nor is dignity often considered at present except in human rights conventions and case law derived from the United Nations Convention on the Rights of Persons with Disabilities^13^ or ECtHR case law concerning torture when it is often considered alongside personhood.^14^

### Methods

The framework we have adopted is to consider the principles of the European Convention on Human Rights (ECHR) in relation to treatment for mental disorders, including treatment without consent, as a means of deriving goals. The ECHR and the judges of the European Court of Human Rights (ECtHR, ‘The Strasbourg Court) are drawn from both common law and Roman law jurisdictions, so that their interpretations and precedents may be informative concerning alternatives to strict application of capacity tests.

### The European convention on human rights

There are many international conventions on human rights, social, cultural, and economic rights, and related matters. The ECHR is taken here as a useful guide to the relationship between neuroethics and treatment without consent as manifested in the laws of many modern states. The ECHR has been accepted by the members of the Council of Europe (CoE, 46 member states, 675 million population), who undertake to be bound by the rulings of the European Court of Human Rights (ECtHR), often referred to as the Strasbourg Court. Central to the ECHR is a system for the rule of law. The ECtHR is widely respected as an authoritative source for interpretive case law and precedent, binding on the member states of the CoE and showing consistency, coherence, and respect for legal process and rules of evidence that is lacking in the interpretative processes of other international conventions and international bodies. The ECHR has also been incorporated into the law of the European Union (EU, 27 member states, 449.2m population) and is now being interpreted alongside the United Nations Convention on the Rights of Persons with Disabilities.^13^

Box 1 **ECHR Article 2:**

Everyone’s right to life shall be protected by law. No one shall be deprived of his life intentionally.….

Two substantive obligations arise from ECHR Article 2 (Box 1): a positive obligation to protect the right to life by law, which in practice requires a regulatory framework, and a negative right, the prohibition of the intentional deprivation of life, with some exceptions, and a positive obligation to prevent suicide, particularly for persons in detention. Those who are vulnerable due to mental disabilities, and those who are detained by law in hospital, require a stricter standard of protection.

Box 2 **ECHR Article 3:**

No one shall be subjected to torture or to inhuman or degrading treatment or punishment.

ECHR Article 3, the prohibition of torture (Box 2), is an unqualified right. Case law of the ECtHR is enlightening: “A measure which is a therapeutic necessity from the point of view of established principles of medicine cannot, in principle, be regarded as inhuman and degrading. The Court must, nevertheless, satisfy itself that a medical necessity has been convincingly shown to exist and that procedural guarantees for the decision exist and are complied with (Jalloh v. Germany [GC], 2006, § 69)”.^14^ The concept of “therapeutic necessity” or “medical necessity” is a widely recognized and essential component of legal and ethical reasoning and decision-making in relation to treatment and consent generally.

Box 3 **ECHR Article 5 § 1 (e) of the Convention provides:**

Everyone has the right to liberty and security of person. No one shall be deprived of his liberty save in the following cases and in accordance with a procedure prescribed by law: […] (e) the lawful detention […] of persons of unsound mind

In accordance with ECHR Article 5, the right to liberty (Box 3), there are three minimum conditions for lawful detention on the basis of unsoundness of mind: (1) the person concerned must reliably be shown to be of unsound mind, that is, a true mental disorder must be established before a competent authority on the basis of objective medical evidence; (2) the mental disorder must be of a kind or degree that warrants compulsory confinement; and (3) the validity of continued confinement must depend upon the persistence of such a disorder.

The Strasbourg Court has held that no deprivation of liberty of a person considered to be of “unsound mind” is permitted if it has been ordered without seeking the opinion of a medical expert, giving evidence that is reliable. Any other approach falls short of the required protection against arbitrariness.

In Winterwerp v. The Netherlands (1979 6301/ 73 ECHR 4, paragraph 37),^15^ the Strasbourg Court held that “the convention does not state what is to be understood by the words ‘person of unsound mind’. This term is not one that can be given a definite interpretation…… it is a term whose meaning is continually evolving as research in psychiatry progresses, and increasing flexibility and treatment is developing and society’s attitude to mental illness changes, in particular so that a greater understanding of the problems of mental patients is becoming more widespread.” This definition may be compared with the definition in the United Nations principles for the protection of persons with mental illness,^16^ principle 4 “determination of mental illness” which emphasizes the importance of international classification systems and definitions such as those of the World Health Organisation International Classification of Mental and Behavioural Disorders.^17^

Notably, there is no requirement that detention on the grounds of unsound mind is exclusively for treatment. Protection of life, bodily integrity, and dignity may also be engaged. However, it is also the case that detention without treatment, for example, delaying treatment after detention pending a second hearing to determine incapacity, is harmful^18^ ^,^^19^ and therefore unethical. Detention without treatment is merely imprisonment.

Box 4 **ECHR Article 8:**

the right to respect for private and family life, home and correspondence…. there shall be no interference by a public authority with the exercise of this right except such as is in accordance with the law and is necessary in a democratic society in the interests of national security, public safety or the economic wellbeing of the country, for the prevention of disorder or crime, for the protection of health or morals, or for the protection of the rights and freedoms of others

ECHR Article 8 sets out the right to private life. The concept of private life covers the physical and moral integrity of the person (X and Y v. the Netherlands, 1985, § 22.)^20^ Emergency medical interventions on life-saving grounds performed in the absence of the patients’ consent are not incompatible with the Convention. An individual’s involvement in the choice of medical care options available and consent to such treatment falls within the scope of Article 8. There is a general requirement on States to provide a legal framework of guidelines and procedures setting out the elements of informed consent and to ensure high professional standards in this area.

The ECtHR has held that mental health must be regarded as a crucial part of private life associated with the aspect of moral integrity. The preservation of mental stability is in that context an indispensable precondition to effective enjoyment of the right to respect for private life (Bensaid v. the United Kingdom, 2001, § 47).^21^ Compulsory medication of a mentally ill patient may be justified in some circumstances, in order to protect the patient and/or others. However, such decisions must be made against the background of clear legal guidelines and with the possibility of judicial review.

Although the right to health is not among the rights guaranteed under the ECHR or its Protocols, Contracting States are under a positive obligation to take appropriate measures to protect the life and health of those within their jurisdiction and there is also an obligation “to place the best interests of the child, and also those of children as a group, at the centre of all decisions affecting their health and development.”

It can reasonably be taken that the “best interests” of persons of unsound mind, and especially those who are vulnerable due to mental disabilities and those who are detained by law in hospitals, should also be at the center of decisions affecting their health, to protect the patient and / or others.

### Principled and process approaches to ethical reasoning about treatment

Legal decision-makers should not impose legal values and priorities (as distinct from lawful processes) imported from the criminal justice system into medical ethics and professional decision-making. For example, Rawls prioritizes liberty above all other values.^22^ Whether Rawls’s primacy of liberty is a principle or merely a conjecture is open to challenge.^23^ ^,^^24^ In the ECHR and other similar systems of human rights, liberty is a qualified right. Medical ethics must be subject to the principles of natural justice, with respect for rights and compliance with the law. But it should not be acceptable to denigrate as “paternalism” the medical obligation to act with compassion in the best interests of a vulnerable and impaired person, either professionally or as a state.

Medical, psychiatric values and priorities in decision-making can be identified with scientific values. Bronowski^25^ pointed out that successful factual scientific discoveries require a commitment to values such as truth, good explanations, and openness to new ideas and change, further entailing tolerance, integrity, and openness to debate. Deutsch^26^ developed a theory of moral reasoning from his scientific emphasis on the importance of “good explanations” in successful science. Good explanations are hard to vary because they are about reality and close to truth (verifiable observations in the real world, consistent with other known scientific explanations and explanatory systems, cannot simply be bent to suit some bias or vested interest), simple or elegant (make no assumptions beyond necessity), are falsifiable (constrained by existing knowledge including other good explanations), and a good explanation reaches out to explain other problems beyond the problems it was created to solve. Further, Deutsch goes on to assert that the same scientific characteristics of good explanations can be used to find solutions to moral problems.^26^ Since the process of solving moral problems is ethics, this is a scientific form of ethics and is likely to be more successful in the real world than the conjectural, literary-critical, or normative approaches.

**Figure 1.**
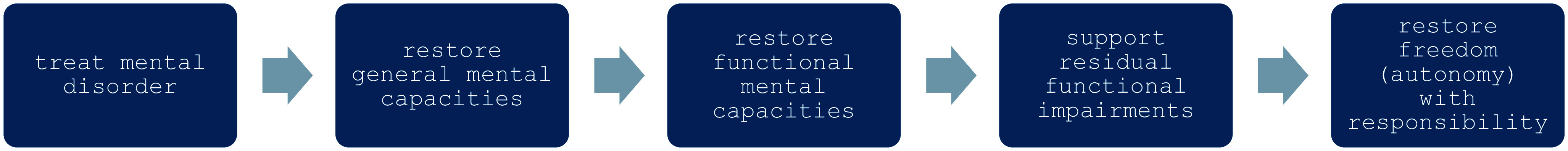
Ethics as process

A compatibilist “process” approach may reconcile many of these apparent clashes between legal and medical decision-making. Figure 1 offers an alternative to Rawls’ hierarchical principles of justice, which insist on freedom before other values and elevates this value to a principle by an act of reification. Rawls’ second principle, that inequality is only to be allowed if the worse off will be better off than they might otherwise be, seems in keeping with the system proposed in Figure 1. In this ethical system, there can be no freedom without responsibility and no responsibility without intact functional mental capacities. Therefore, the first step and first priority is to treat mental disorders with a view to restoring capacity, thereby restoring responsibility, to whatever extent is possible. The alleviation of suffering and restoration of dignity in the best interests of the person concerned are inseparable benefits and measurable health gains from this process.

### Goals derived from principles

We propose that goals can be derived from these principles, and we propose also that goals can be ordered according to a rational process. This contrasts with principles, which are universally agreed but extremely difficult to resolve into an ordered hierarchy for decision-making. We propose that the overarching goal of treatment in neuropsychiatry is to achieve health gains for patients. This process starts with the obligation to ensure that all rights are vindicated and legal processes are complied with. Next is the duty of care to ensure a safe, violence-free therapeutic environment for patients and clinicians. This enables the next stage in a process of goal-directed activity to prioritize effective treatments over other activities. The fourth stage in the process is the evaluation of health gains achieved and the duty to continuously improve health gains through research, development, and evaluation of treatment at the individual and organizational level for specific cohorts and general populations.

## Processes

Clinical decisions of great consequence are made regarding patients with severe mental illnesses. Such decisions should be made in accordance with an ethical process to ensure fairness, the right to be heard, consistency, proportionality, respect for rights, and compliance with law. The obligation to make such decisions ethically is all the greater where the patient is vulnerable and may lack the ability to make competent decisions for themselves. Among the most consequential and complex decisions are those regarding deprivation of liberty and triage to levels of care, including levels of therapeutic security,^27^ ^–^^29^ the management of waiting lists,^30^ decisions about length of stay,^31^ ^,^^32^ and restoration of competency, conditional discharge,^33^ and community treatment orders,^34^ or the use of restrictive practices such as seclusion or restraint.^35^ There is increasing evidence that such decisions are more reliable when a structured professional judgment instrument is used to guide the clinical assessment and decision-maker,^36^ ^–^^39^ and when such structured professional judgments are considered within a decision-making judgment support framework such as an admissions panel, court process, or review board.^40^

### Diagnosis as an ethical process in psychiatry

Establishing a diagnosis is often the first step in a process leading to treatment. The correct process of establishing a diagnosis has consequences for the evaluation of prognosis. This in turn has a large influence on decisions about the benefits and risks of treatment. The operational diagnostic criteria of the American Psychiatric Association’s Diagnostic and Statistical Manual (APA DSM)^41^ and the World Health Organisation’s International Classification of Diseases for Mental and Behavioural Disorders (WHO ICD^17^) favor a syndromal approach with operationalized criteria that works well with palliative and ameliorative treatments. The eventual emergence of disease-modifying treatments for causes will fracture these diagnostic categories. This is not to be taken as indicating the end of diagnosis.^42^ Diagnosis will remain essential for assessment of prognosis and selection of treatment.^43^ ^,^^44^

Recent progress in the assessment and diagnosis of neurodegenerative disease with perceptual and processing disturbances (NDPPD) has focused on staging these “process” illnesses, first characterized as dementia praecox because the onset is often with decline in functional ability in late adolescence before the emergence of delusions and hallucinations sufficient to meet diagnostic criteria for schizophrenia.^45^ Stage “zero” or at-risk mental state, has a predictive power for the emergence of any of the NDPPD mental illnesses.^46^ ^,^^47^ This is often bound up with substance misuse, particularly cannabis.^48^ ^,^^49^

A “chicken and egg” conundrum of causation arises from the circular relationship between vulnerability to substance misuse due to NDPPD (for example, schizophrenia), while early onset and severity of substance misuse also leads to the emergence of NDPPD with delusions, hallucinations, and cognitive decline, with a consensus in favor of cannabis as a causal agent.^48^ ^–^^52^

Hodgens^53^ ^–^^55^ described young people with early onset of conduct disorder and substance misuse who have single or brief episodes of psychosis resembling schizophrenia with full remission of symptoms and full functional recovery after a single episode or between episodes. These are probably best described as psychoactive substance-induced psychoses because they are distinct from Kraepelin’s dementia praecox and “process” schizophrenia. These are notable because they have good outcomes.^43^ Other patients develop the Kraepelinian core syndrome: they typically have had no prior conduct disorder or substance misuse and develop schizophrenia with a relapsing and remitting course, progressing from stage zero (at-risk mental states) to stage 1 (a single episode with full recovery) through stage 2 (relapsing and remitting with full recovery between episodes), stage 3 (relapsing and remitting with some residual symptoms and functional impairment between episodes, gradually deteriorating over time), and stage 4 (treatment-resistant symptoms and severe functional impairment).^56^ ^,^^57^ In this context, the benefits of preventing relapses^18^ ^,^^19^ ^,^^58^ become essential for making informed decisions about treatment, particularly the use of long-acting injections and long-term treatment to prevent relapses and progression.

### A critique of capacity based tests

It is sometimes suggested that the requirement for a medically diagnosed mental disorder could be dispensed with so that legal processes could rely solely on functional mental capacity.^59^ ^,^^60^ Assessing mental capacities to reach an opinion about a functional mental capacity is inherently a circular, self-referential process. The capacities to understand, or to reason, or to appreciate and to communicate are evident only at the level of consciousness (a high order of emergent complexity); therefore, the assessed functional mental capacity (which is at the same level of emergent complexity) is circularly related to the end assessment. Insisting on a “functional” test does not solve this circularity. Requiring an antecedent test of impairment or dysfunction at more objective “microscopic” levels of emergence—neurophysiological, neuropathological, and neuropsychological—is necessary, if only as a protection against malingering, and is also necessary as a protection against many forms of bias and error due to mind-body dualism—the impossibility of distinguishing between “will not” and “cannot.” A higher-level emergent phenomenon such as consciousness is never more than the sum of the interactions and organization of lower-level entities such as normal and abnormal anatomy and physiology and the intermediate level of neuropsychology or psychopathology. Whether all properties of an emergent system can at present be explained in terms of an underlying constituent part or not, all of the known pathological processes affecting consciousness (anesthetics, intoxicants, injuries, epileptic phenomena, cerebrovascular phenomena, metabolic phenomena, neurodegenerative diseases, developmental disorders, mental diseases, and impairments of mental capacities) have been pathologies of constituent, underlying, “smaller scale” parts and systems (genetic defects, biochemical process dysfunctions, anatomical lesions, growths, and injuries). And all effective treatments (antibiotics, neuroreceptor agonists or antagonists, immunomodulators, and immunosuppressants) have been active at the level of lower-level constituent parts. By contrast, mind-body dualist approaches and “strong emergent” theories of mental disorders and incapacities depend on circular reasoning and on the reification of notions (mistaking the name of an idea for a real thing, for example, ego, id, and super-ego) that cannot be demonstrated or falsified.^61^ Treatments based only on mind-body dualism cannot show any measurable benefit beyond the placebo effect.^62^ ^–^^64^

There are other approaches to the ethical priorities that should be considered and weighed in relation to competence to make decisions of greater consequence. The loss of human dignity and the suffering from tormenting symptoms can be effectively treated and should not be left untreated by a caring society or a conscientious, compassionate clinician. The willingness to intervene and use restrictive means to achieve health gains—alleviating suffering due to symptoms, restoring dignity through enhanced functional ability, restoring civil autonomy and responsibility, fostering recovery, and hope are necessary professional and ethical values for doctors, nurses, and other health professionals. Acting in the best interests of vulnerable persons of unsound mind is a primary ethical obligation for doctors, nurses, and allied health professionals. Avoiding the medical necessity to intervene out of scruples for legal values and priorities (as distinct from lawful processes) falls short of medical professional ethics.

The legal exclusion of family members from the processes of making decisions for incapacitated adults is one of the least democratic, least supported pieces of legal innovation of recent decades. There is little democratic support for this and much unhappiness among families and carers.

### Legal processes

Precedent setting judgments have interpreted legal provisions for detention and treatment under mental health law as “purposive” and “designed for the protection of the citizen and for the promotion of the common good”.^65^ Any resiling from this position would be regressive. The goal of any detention due to medical necessity must be to deliver effective medical care and treatment. Detention without treatment is merely imprisonment. For example, legal structures that allow detention under mental health legislation while delaying treatment until a court hearing at some later date to determine incapacity and permit treatment without consent should be regarded as self-defeating and unnecessary delays to essential treatment. Delayed treatment and deliberately prolonged duration of untreated psychosis are now known to be seriously harmful.^18^ ^,^^19^ Such unmedical and harmful delays are therefore unethical processes. This unethical delay is a good example of prioritizing liberty as a principle while failing to recognize the process and goal-directed necessity of treating to restore mental health and capacity in order to enjoy the restoration of free will, responsibility, and liberty.

Council of Europe recommendation number REC (2004) 10 is derived from the case law of the ECtHR.^66^ It is often regarded as a “model mental health act,” and its language has found its way into many of the mental health acts of European states.^13^ It defined “treatment” as an intervention (physical or psychological) on a person with a mental disorder that, “taking into account the person’s social dimension, has a therapeutic purpose in relation to that mental disorder.” Treatment may include measures to improve the social dimension of a person’s life, and “therapeutic purposes” includes prevention, diagnosis, control, or cure of the disorder, and rehabilitation.

### Medical necessity

The following is an ordinal scale (Box 5) intended to allow decision makers to weigh up “medical necessity” as a justification for intrusive or restrictive practices or compulsion—including compulsory detention. These “weights” should be considered in the balance against the parallel scale assigning weights to the possible adverse consequences (Box 6). Examples might include nasogastric feeding in life-threatening anorexia nervosa and other forms of compulsory medication, including by intramuscular injection or in rare cases, by nasogastric tube. The scale ranges from the immediate need to preserve life (arguably a right under international covenants on human rights) through to the necessity of treatment in order to restore dignity and mental capacity (according to the principle of reciprocity—no deprivation of liberty without providing treatment necessary to restore autonomy or dignity) to necessity because without treatment the right to enjoy the full potential of life may be lost.

It must be emphasized that any form of intrusive, restrictive, or compulsory intervention without consent can be justified only where the person concerned lacks the functional mental capacity to give or withhold consent. Therefore, any such intervention without consent must end when capacity is restored. In practice, however, such intrusive and restrictive interventions should always be ended earlier than the restoration of capacity on recovery and safety grounds. The prevention of imminent and serious violence to others, preserving the therapeutic milieu for all patients, and exercising the duty to keep vulnerable patients safe may also be invoked as a medical necessity.^67^

Interesting questions arise concerning the balancing of the rights of staff not to be assaulted while caring for violent patients and the necessity of preserving therapeutic relationships.^68^ Medical necessity may in some circumstances be taken as a reason to intervene and treat without consent, whether there is capacity or not. In such cases, a hierarchy of necessities and a hierarchy of consequential risks are to be weighed in the balance.

Box 5 **Medical Neccessity as an ordinal scale:**

10 = necessary to save life (urgency is essential here—the doctor must act at once or as soon as possible, eg, refusal of all fluids)

09 = necessary to preserve life (here the intervention is necessary because otherwise deterioration to a life-threatening state will be certain, and within days. This can apply also to some maintenance treatments)

08 = necessary to prevent irreversible and serious physical injury to the patient (“serious” here would mean a disability that would alter the person or personality of the patient leading to a permanent physical or mental disability. This echoes a definition of degrading treatment that might alter the character of a person)

07 = necessary to alleviate suffering that is serious and degrading for the person concerned.

06 = necessary to bring to an end the use of other compulsory restrictive practices that might otherwise continue indefinitely where such practices are themselves a cause of suffering or are degrading (eg, long-term isolation, long-term restraint)

05 = necessary to prevent injury to others and therefore to facilitate resocialization and integration into social contact with others (interesting questions arise here concerning the rights of nursing and care staff. Some patients may injure others because they prefer to be isolated and do not experience this as suffering or degradation and so fall outside the definitions in 06 and 07).

04 = necessary to restore functional mental capacity.

03 = necessary to achieve the least restrictive legal and social situation—either to be discharged from detention or to move to a minimally restrictive regime.

02 = necessary to restore or preserve dignity. For example, a patient with behavior that makes socialization difficult might benefit from treatment to enable resocialization and integration with others. (Some patients repeatedly scream, strip, rush about, intrude on others….)

01 = necessary to achieve and enjoy full potential in life. For example, a person may on achieving remission gain a much better quality of life in ways beyond the preservation of life or alleviation of suffering. Self-actualization through creativity, self-transcendence through meaningful socialization, regaining hope—these are all potential benefits of clozapine (and some other) treatments.

Box 6 **Adverse consequences as an ordinal scale:**

10 = restraint and procedures (eg, passing of a nasogastric tube) may be fatal due to restraint asphyxia or cardiac events during a struggle.

09 = misadventure may occur (eg, aspiration pneumonia may occur due to an incorrectly placed NG tube)

08 = known side effects of the intervention (eg, ECT, clozapine, or other medication) may be serious though rare, and in most cases treatable (anesthetic adverse events, agranulocytosis, pulmonary embolism, megacolon….)

07 = the procedure may itself cause pain and gagging

06 = the procedure may itself cause suffering due to humiliation and shame

05 = others may be injured in the course of the procedure itself. This may include exposure of clinicians to biohazards

04 = eventual engagement in a process of informed consent and therapeutic engagement may be made more difficult due to the alienating effect of the intervention.

03 = other processes of therapeutic engagement and negotiation may be prevented or inhibited while the process is ongoing and subsequently.

02 = the procedure may be inherently undignified while it is happening

01 = the procedure may be experienced as traumatic, and this may shape long-term cognitive sets and perceptual biases concerning mental health treatments in a way that is against the person’s best interests.

### Consent to treatment

Functional mental capacity is not the only way to assess competence, nor is it the sole consideration even in jurisdictions that prioritize it.^69^ Psychiatrists are accustomed to assessing the functional mental capacity to give consent to treatment. We are accustomed also to the need to ensure that consent is obtained free of duress and on the basis of having fully informed the patient of the benefits and risks of the available treatment choices. Box 7 sets out the most widely accepted criteria for assessing functional mental capacity to give consent to treatment.

Box 7 Understanding

Reasoning—comparative and consequential

Appreciation

Communicating a decision

The problem with legal reasoning about functional mental capacity is the legal belief that different functional mental capacities are independent of each other. A person who lacks the functional mental capacity to give consent to treatment for a psychosis may nonetheless be held to have the functional mental capacity to make a will or to marry. While it is reasonable to accept this when the decisions concerned are different in complexity, legal reasoning often appears to be based on mind-body dualism and a naïve acceptance of expressed will and preference, though only where this accords with the legal decision maker’s own beliefs.

### Variable thresholds for capacity and competence: complexity and information overload

The difficulty arises that to give more than basic amounts of information is often enough to render the severely mentally ill patient unable to make a choice or communicate a decision.^70^ ^–^^72^ Complex decisions are often beyond the functional mental capacities of the severely mentally ill, while relatively simple decisions are within mental capacities to understand, reason, appreciate, and communicate a decision. Lawyers attempt to gloss over this difficulty by saying that even a fleeting period of apparent capacity may allow a competent decision to be made, even if later the person makes a different choice or repeatedly changes their decision. This appears to the psychiatrist to be legal casuistry when all such fleeting decisions should be regarded as the products of an unstable and unreliable mental state. ECHR Article 8 has been interpreted to mean that the preservation of mental stability is an indispensable precondition to effective enjoyment of the right to respect for private life (Bensaid v. the United Kingdom, 2001, § 47).^21^ Indeed, there is a trend among legal decision-makers to accept this and instead accept “will and preference,” whether capacitous or not, if it is reasonably stable, consistent with personality and preferences when well, and of no serious or life-changing consequence.

### Variable thresholds for capacity and competence: seriousness of consequences

The more serious the consequences of the decision, the higher the level of functional mental capacity that should be required. For this reason, Applebaum and his group were careful to avoid stating any threshold or cutoff score above or below which a patient did or did not have functional mental capacity. Our group offered sensitivity and specificity threshold scores for a “standardised” test (three choices, one of which is “do nothing; two pieces of information for and two against each option”), but in practice this cannot easily be extrapolated to other more complex real-life decisions about mental health.^73^

The Common Law approach privileges a particular set of common law precedents regarding functional mental capacity. The use of Appelbaum’s formulation of functional mental capacity in terms of understanding, reasoning, and appreciation^74^ and the related development and validation of the Mac-CAT on this basis,^75^ ^,^^76^ are correctly regarded as the gold standard in the research literature. This is derived from the Common Law concept of “mind” as a collection of capacities.^8^ This is reductive in order to eliminate the phenomenologically mysterious and subjective aspects of consciousness. These functional capacities are available for clinical examination and judicial consideration.

The Common Law approach to functional mental capacities has limitations. The Mac-CAT family of structured professional judgment instruments for assessing capacity to consent to treatment or to consent to research is demanding. A large proportion of mentally ill persons in hospitals is unable to complete the assessment and is excluded from research samples.^70^ ^,^^72^ This problem is compounded by the relative simplicity of the test task—having to retain two items of information about the advantages of a proposed treatment and two items of information about the disadvantages or side effects, repeated for three options. When extra information is given, an even larger proportion of mentally ill persons are unable to complete the task.^72^ So for “real world” complex choices and decisions faced by clinicians and their severely mentally ill patients, the Mac-CAT instruments are limited in the extent to which their research validation can be generalized.

A method combining interviews where possible and observation in all cases based on Appelbaum’s formulation (understanding, reasoning, appreciating, and communicating a choice) has the advantage of greater inclusivity because observational rating scales can be completed on all patients no matter how disturbed.^36^ This is both ethically more inclusive and scientifically more valid because it does not exclude a large proportion of patients who are too ill (too functionally impaired) to complete the interview assessment.^36^

The basic concept of functional mental capacity is not the only approach to protecting the rights of the mentally ill or cognitively impaired person. The Roman Law concept of “imputability” is derived from a less reductive concept of mind. This recognizes that a mental illness resulting in delusions, hallucinations, thought disorder, and other changes in neuropsychological function,^77^ ^–^^80^ metacognition,^81^ and moral reasoning^82^ ^,^^83^ may all impact and impair competence to make decisions about one’s person and health, welfare and dignity, estate and finances, and capacity to form specific intentions.

Elsewhere, Carrara has argued^1^ ^,^^2^ that any impairment of consciousness is an impairment of mental capacity to make responsible decisions that is generalizable and not function-specific. This is in keeping with modern neurophysiology and neuropsychology and contrary to the implicit mind-body dualism of legal concepts of functional mental capacity.

A layered concept of consciousness, from the neurophysiological to the neuropsychological and from the neuropsychological to personality or symptoms of dysfunction such as delusions, may allow a more correct assessment of capacity to make competent decisions, in the same way that “imputability” allows a different approach to criminal responsibility. The difficulty is then to set the threshold for legal incompetence or diminished responsibility. For example, it is unlikely that personality could amount to an impairment of consciousness that mitigated or reduced either the capacity to make decisions or the responsibility for criminal acts. Kenny’s critique of circular reasoning and nominalism (reification) as false justification for such opinions regarding personality and responsibility is helpful here.^4^ ^,^ ^6^ Kenny illustrates his view with an account of a case law precedent in which a defendant was convicted of causing death by witchcraft.^7^

## Treatment

The overarching goal of treatment is to achieve health gains for patients. Saving life and prolonging life while enhancing quality of life are implicit within this. Health gains can be measured under four broad categories of “recovery”: symptomatic recovery and the duty to prevent suffering; functional recovery and the obligation to restore or enable as much self-determination, independence, and choice as possible within the person’s abilities; civil recovery of the ability to exercise legal autonomy and responsibility; and personal recovery, the ability to produce or co-produce one’s own care and treatment plan, to have one’s voice heard, and to have hope.^34^

The goal of achieving health gains may have been achieved at one time through conservative measures, waiting and supporting; in long-term care for untreatable or progressively degenerative diseases, through ensuring quality of life, dignity and providing the “scaffolding” of supportive care for basic biological functions and activities of daily living to ensure dignity, sensitivity to preferences in the absence of capacity, and freedom from symptomatic suffering. At its highest level, this structured care and treatment can facilitate higher functions such as self-actualization (the expression of the self as a person) and self-transcendence (the experience of being an active part of one’s family and community).

In modern times, treatment in oncology, cardiology, and respiratory medicine has led to ever-improving 5-year survivals and lengthening life expectancy. During the same period, standardized mortality ratios have progressively worsened for NDPPDs such as schizophrenia and bipolar affective disorder,^84^ with a measured 16-years loss of life expectancy in NDPPD.^85^ ^–^^87^ Health gains such as life expectancy and the four recoveries, symptomatic, functional, civil, and personal recovery are as important in psychiatry as they are in any other area of medicine.

### Variable thresholds and the effectiveness of treatments: palliative, ameliorative, and disease-modifying treatments

All treatment decisions involve weighing the consequences of untreated disease, the benefits of treatment, and the possible side effects of treatment, then making a decision in the balance. It is the responsibility of the patient to make the decision, but there is a strong professional responsibility on the doctor to give the advice needed for an informed decision.

Some treatments are more effective than others. In psychiatry and medicine generally, treatments can be divided into palliative, ameliorative, and disease-modifying. Palliative treatments do no more than alleviate troublesome symptoms and suffering. Ameliorative treatments may interrupt or delay a disease process for example, excising a tumor to relieve obstruction or using a diuretic to relieve congestive cardiac failure. Disease-modifying treatments act on the causative pathological process. For example, treatments in oncology can be divided into palliative treatments, for example, pain relief; ameliorative treatments, such surgery to prevent bowel obstruction; and disease-modifying treatments, for example, surgical excision, radiotherapy, and chemotherapy combined with monoclonal antibodies to target cancerous cells. In rheumatology, there has been a dramatic change from palliative and ameliorative drugs—non-steroidal anti-inflammatory and immunosuppressive drugs to disease-modifying drugs^88^ particularly biological agents.^89^ Loss of hand function due to rheumatoid arthritis is now seldom seen in developed countries. Similar progress with disease-modifying treatment has occurred in Crohn’s disease, ulcerative colitis, Sjogren’s syndrome, and other inflammatory diseases. In congestive cardiac failure, diuretics, vasodilators, and surgery will have ameliorative effects on the secondary pathophysiology of a failing heart, but only treatments that address causes will have disease-modifying effects, for example, cardiac valve replacement when the cause is stenosis or regurgitation.

At present, almost all treatments available for NDPPD are palliative or ameliorative, alleviating the secondary effects of disease processes (symptoms, suffering) and possibly ameliorating patterns of relapse, but with no unequivocal disease-modifying effects on the earliest and most disabling stages of neurocognitive impairment and later cognitive decline.^90^ However, there is emerging evidence that longer duration of untreated first-episode psychosis predicts poor long-term outcomes with worse symptoms, impaired global function, and neurocognitive abilities.^18^ ^,^^19^ It follows that earlier intervention and the use of relapse-preventing medicines, such as long-acting injections of receptor-blocking medication are disease-modifying. The long-term prospective studies concerning the duration of untreated relapses (second and subsequent episodes) are not available,^18^ ^,^^19^ but a relevant finding is that the benefit of treatment to prevent relapses (long-acting injections of receptor-blocking medication) is sustained over very long periods, with relapses occurring after discontinuation as much as 8 years later.^58^

The balancing of risks and benefits will be very different for a disease that is inconvenient or uncomfortable when compared to a disease that causes suffering, is life-shortening or disabling. We must now recognize that NDDPDs such as schizophrenia and bipolar disorder are life-shortening diseases^87^ as well as disabling and causes of suffering. The additional consideration arises that the threshold for intervening without consent will vary according to the risks and effectiveness of the treatment, even when this treatment is long-term use of long-acting injections of antipsychotic medication.^58^ ^,^^87^ ^,^^91^ ^,^^92^

### Disease-modifying treatment and variable thresholds for early intervention when treating without consent

Whether or not early intervention with the treatments currently available can prevent progression of NDPPDs such as schizophrenia, it is foreseeable that disease-modifying treatments will soon be available. Such treatments are emerging for Alzheimer’s disease and some genetically determined neurological disorders. Similar treatments may emerge for the early stages of neurocognitive decline in adolescents with early-stage schizophrenia. This will present a different set of ethical considerations when deciding when to offer voluntary treatment and what the information and advice should be that constitutes informed consent.

The ethical justification for treatment without consent where the treatment is disease-modifying may be more in favor of early intervention the stronger the evidence is for disease modification. This raises the question of “medical necessity,” a necessary condition for many forms of treatment or therapeutic interventions without consent in those who lack functional mental capacity or general mental capacity due to severe mental illnesses or other forms of mental disorder^14^ Box 5. Whether a disease-modifying treatment that addresses loss of life expectancy, prevents impairment of, or restores functional mental capacities can be interpreted as a medical necessity or a lesser justification for treatment without consent will probably require legal adjudication and precedent.

One candidate for disease-modifying treatment in NDPPD may be the permanent editing of genes (CRISPR). Since illnesses such as schizophrenia appear to be polygenic in predisposition, this may not be a way forward, though a greater concentration on the genes responsible for early neurocognitive decline may lead to interventions in the earliest stages of disease. Any permanent change in a gene that is vital to some aspect of neurocognitive function may be seen as a permanent change to the personality, temperament, or disposition. This will raise ethical problems since treatments that cause permanent changes to personality may be interpreted as inhuman, degrading, or even dehumanizing, although there is a complex counterbalance concerning medical necessity.^14^ Epigenetic editing by switching genes on and off reversibly is more likely to be an ethically acceptable disease-modifying treatment.

## Evaluation

There is an ethical duty to evaluate the outcomes of treatments and the delivery of treatments in models of care more generally.^93^ This is essential since otherwise goals that were set in order to ensure that ethical principles are observed may be taken for granted. The aphorism “if you can’t count it, you can’t see it, and if you can’t count it, it may not be real” applies here. There is an additional obligation in medical values to ensure that outcomes and health gains for patients are continuously improved through the virtuous circle of excellence: research, development, teaching, and training.^94^

### Principled ethical barriers to research progress in psychiatry

In research on interventions that have low or negligible levels of medical necessity as described in Box 5, there are inevitably no adverse consequences as described in Box 6 and it is sufficient to rely on “will and preference” as a standard for competence to consent, whether a functional mental capacity test can be completed or not. But these “low necessity” interventions are generally either placebos or at best palliative, seldom ameliorative, and never disease-modifying.

Psychiatry has much to learn from oncology. The success of oncology arises from the systematic organization of international multicenter randomized controlled trials in which “treatment as usual” is compared to “treatment as usual plus.” The result has been steady, incremental improvement in outcomes year by year. This has not yet happened in psychiatry.^94^ ^–^^97^

Due to ethical reliance on tests of functional mental capacity, research on treatment usually excludes all those who lack functional mental capacity to consent and all those who are detained under mental health legislation due to the obvious implied duress inherent in such a situation. Research on treatment is therefore biased by excluding the most severely ill. Excluding the most severely mentally ill is an ethical error, unfairly depriving them of the benefits of the best research. The result is not just lack of progress but worsening standardized mortality ratios and worsening 5-year survivals.^11^ We do not know of any published attempt to examine and weigh the arguments for and against these conflicting ethical considerations.

In the absence of any prospect of randomized controlled trial research leading to incremental improvement, we are obliged to rely on prospective observational cohort studies, which should be inclusive and naturalistic. These can be powerful and informative. We are also obliged to wait for step-wise “break through” scientific progress. For example, if new biological treatments prove to be effective in Alzheimer’s disease or multiple sclerosis, then NDPPD, such as schizophrenia may be considered as possible additional beneficiaries if enough is known about the underlying pathophysiology to infer similarity. Inflammatory bowel diseases now benefit from the biological disease-modifying treatments for rheumatoid arthritis.^98^ This approach is, however, inherently slower and less productive.

An unfortunate consequence of the right to privacy has been the burdens imposed on access to population data for medical research. The Scandinavian countries have shown the enormous advantages for progress in diagnosis and treatment from ensuring that the right to data privacy is qualified and limited for the purposes of medical research.^99^ ^–^^101^

## Conclusions: Beyond paternalism and best interests

Characterizing as “paternalism” the compassionate duty to intervene actively in the best interests of a mentally disordered person is a pejorative rhetorical device. There is a duty to intervene with effective treatments that restore functional mental capacities, restore clear consciousness, awareness, and reality testing, restoring free will, responsibility, and autonomy. To refrain from intervening because of Rawls’ theory-based prioritizing of liberty over all other principles or processes would be contrary to scientific realism, medical values, and medical ethics.

There is a difference between violence (coercion) and force, according to Hannah Arendt. Violence is destructive, even when it is state-authorized; force can be constructive, transforming routine and repetitive labor into creative and lasting work. Arendt held that it should be possible to use the power of the state as a form of force creatively and not as a vehicle of violence, which can only be destructive.^102^ ^–^^104^ For example, it is methodologically possible to show that restrictive interventions are used proportionately and in accordance with law in a psychiatric intensive care setting to prevent imminent violence.^35^

An analogy can be drawn from Schrodinger’s account of the distinguishing features of the living cell, from chaos to order, from order to disorder and decline into entropy and death.^105^ In psychopathology and phenomenology, mental illness is destructive by its pathological nature. Judgment is impaired so that bad decisions are made, with outcomes that are against the best interests of the person concerned. But at a remove, what is happening is that the accumulated information and wisdom of the developed adult, the ability to adapt to the physical and social environment, is dissipated in entropy. Organization and systems that make the conscious personality, the integration of appetites and impulses with the deliberative restraint of long-term goals, are coarsened and degraded in fear and anger, shame and disgust,^106^ entropy and chaos.

In the psychiatric treatment of delirium, psychosis, mania, or severe depression, treatments are effective if they restore the pre-existing organized and developed personality and consciousness—this is more than the simple restoration of some level of functional capacity, though it may require as a first step the restoration of general mental capacity, or at least the partial restoration, or the halting of further mental disintegration. Re-conceptualizing all of this as a process of opposing entropy with organization, confusion with perception, metacognitive errors with reason, and disinformation with information offers a conceptual structure with which to reason ethically about the use of legal compulsion (Arendt’s “force”) to prevent violence and reverse destruction.

### Future directions

At the beginning of this essay, the capacity-based approach to legal concepts of mind in common law jurisprudence and forensic psychiatry was noted as distinct from Carrara’s approach based on layered consciousness, described elsewhere in this collection. The connection between consciousness, agency, and free will as described in that system corresponds more closely with the Roman law concept of imputability. The system of layered consciousness described by Carrara is firmly embodied in neuroscience, anatomy, neurophysiology, and epigenetics. Carrara’s system allows an approach to higher functions, including personality and identity. The two systems are therefore completely compatible at the level of embodiment and amenable to scientific modeling and hypothesis testing in the clinic as well as the laboratory, at least at the embodied levels.

Scientific theories cannot be proven or disproven in the courts, although courts can ignore science if they wish. Courts may accept non-scientific expert evidence because of the current or local social perception of cultural and artistic merit. Courts may accept expert evidence based on mind-body dualism and the related beliefs of postmodernism, critical theory, and denial of concepts of truth and fact in social discourse or even in science. Such non-scientific approaches will not sustain the dignity and respect due to the law, and such non-scientific approaches generally fall short of sustaining the dignity of the severely mentally ill person.

A system of laws concerning mental health, autonomy, and the law should be subject to critical review concerning its values, the elevation of values to principles, and subsequent prioritizing of principles. We prefer the scientific values of Bronowski^25^ and Deutsch^26^ and the ethical process described in Figure 1. There can be no individual liberties without personal mental health. There can be no practice of medicine without the ethical principle of best interests. Dignity should be given prime position among the rights and values. Respect for human dignity demands compassionate social responsibility for our neighbors and fellow citizens.

Carrara’s emphasis on the importance of consciousness as evidence of competence or impairment appears more robust than a narrow approach to functional mental capacity. Capacity, whether general or functional, remains amenable to rules of evidence and legal judgment at the expense of increasingly excessive simplification and circularity. Carrara’s emphasis on the inherent dignity of the person as a neuroethical guide to the use of treatment without consent appears most in keeping with modern human rights principles.

## Data Availability

No personal data were processed in the preparation of this work.
